# Supplementary material for: Visual Analysis and Detection of Contrails in Aircraft Engine Simulations
Source: IEEE Trans Vis Comput Graph. Author manuscript; Available in PMC 2023 Nov 2. (PMC10621327; doi:10.1109/TVCG.2022.3209356)
Supplement: supplementary_material [file NIHMS1938446-supplement-supplementary_material.pdf]

Supplemental Materials
Visual Analysis and Detection of Contrails in Aircraft Engine Simulations

N. Nipu et al.

July 1, 2022

1 Example Visualizations in Expert Workflow

Example plots generated by the CFD domain experts as they attempted to characterize the contrail structures.

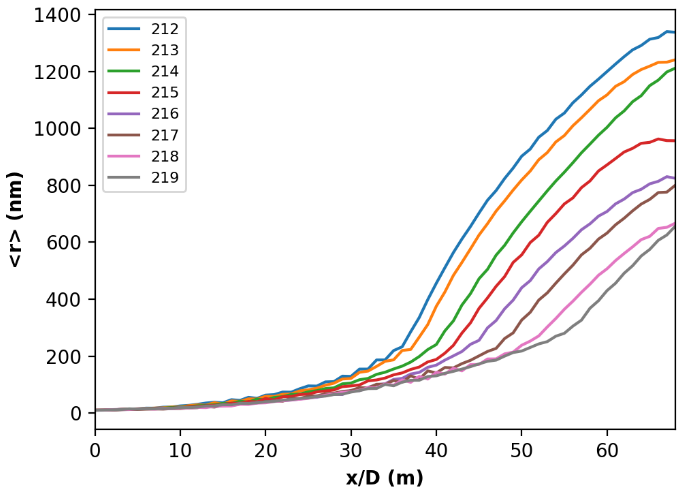

Figure 1: Atmospheric temperature is among the main parameters influencing contrail formation. The plot presents the time-averaged mean radius of the particle as a function of axial distance from the jet, for eight temperatures. Ice particles grow larger where the ambient temperature is lower.

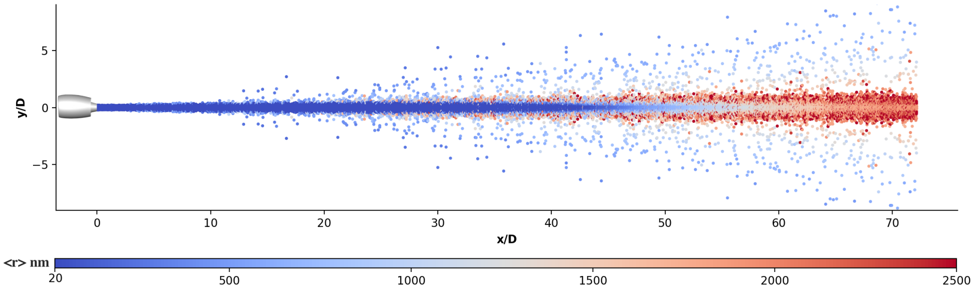

Figure 2: Particle distribution in the computational domain. No ice has formed within the first 15 diameters axial distance from the jet exit, as the plume is too hot for the ice to form. The particles follow the plume's conical shape and have their highest growth on the edge of the plume, where the rapid heat transfer between the jet exhaust and ambient air happens.

2 Input, Model, and Output Parameters

Table 1: Input and Model Parameter Definitions

**Attribute Name**	**Attribute Type**	**Description**
Aircraft Engine Type	Categorical	The type of aircraft engine used for the simulation (e.g., one-stream, two-stream)
Grid	Categorical	Grid used at the wall (e.g., coarse, medium, fine)
Geometry	Categorical	The geometry used for the aircraft (e.g., short, cowl, nozzle)
Scope	Categorical	Nozzle (2D) or Whole airplane (3D)
Turbulence model	Categorical	Model used for engine turbulence
Solution	Categorical	How the particles and fluid flow interact with each other (e.g., coupled, uncoupled)
Boundary Conditions (for Temperature, Pressure, Velocity, Kinetic Energy, Density)	Categorical	Input conditions for different attributes related to simulation runs (e.g., inlet, engine, outlet, farfield, nozzle, turbine, wall, wedgeFront, wedgeBack, zeroGradient, freestream, noSlip, totalTemperature, totalPressure, waveTransmissive, freestreamPressure, kqRWallFunction)

Table 2: Ensemble Output Definitions

Attribute Name	Attribute Type	Description
Position	Numerical	Position of the particles
Temperature	Numerical	Temperature of the particles
Diameter	Numerical	Diameter of the particles
Ice label	Boolean	Ice information of the particles (1 if particle turned into ice, 0 otherwise)

Table 3: Contrail Attribute Definitions

Attribute Name	Attribute Type	Description
Length	Numerical	Length of the contrail structure
Mass	Numerical	Total mass of the ice particles
Mean Temperature	Numerical	Mean temperature of all ice particles for a time point in a simulation run
Number of Ice Particles	Numerical	The total number of ice particles for a time point in a simulation run

3 I/O Panel Design

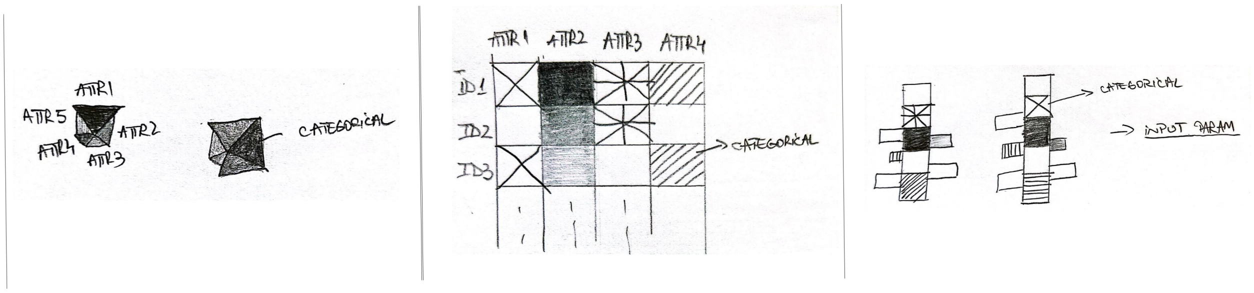

Figure 3: Three low-fidelity prototypes (from a set of ten) for encoding input parameters.

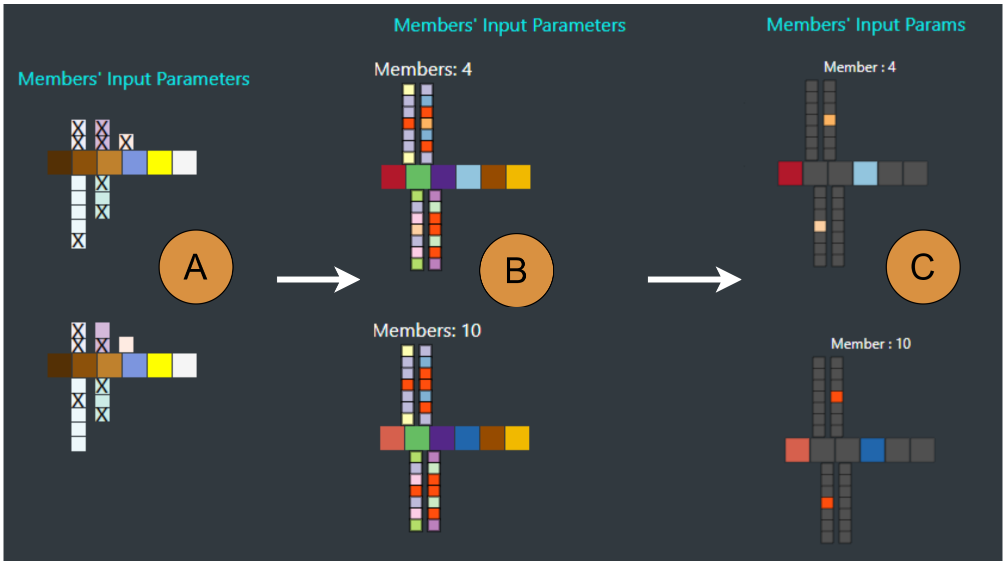

Figure 4: Three hi-fidelity iterations for the colored-tile glyph.

3.1 Encoding Input Parameters

Visualizing a large set of categorical values proved to be a challenging task. Early iterations tried to mix longitudinal and categorical data encodings, including for mixes of input, model, and output parameters (a small subset is shown in Fig. 3). The results suffered from scalability and cognitive load issues. Moreover, the domain experts wished for a clear distinction between the input, model, and output parameter categories.

Based on domain expert feedback, we converged towards a colored-tile glyph (Fig. 4.A), with input variables mapped horizontally, and model parameters mapped vertically. A second iteration further leveraged color maps, and also offset the bottom and top wings, to clarify the distinction among different conditions (Fig. 4.B). The result was overwhelming in terms of cognitive load, even though a color legend was provided and visible at all times. A last, successful iteration (Fig. 4.C) takes advantage of the reduced variability in input parameter values across simulation runs. This streamlined encoding allows the domain experts to distinguish parameter differences between groups of similar ensemble members, rather than among all individual members.

3.2 Encoding Output Parameters

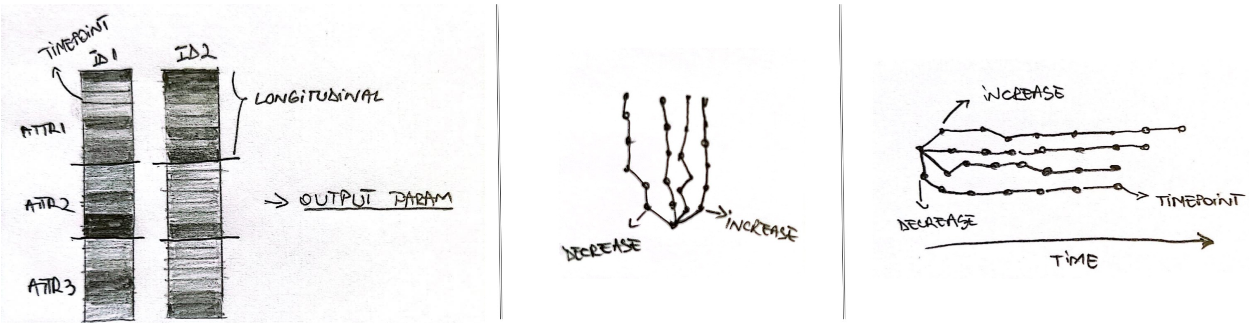

Figure 5: A subset of low-fidelity prototypes for encoding output Parameters.

The output parameter data is longitudinal, and has a variable number of attributes and of number of time stamps. The design process explored a wide range of possible temporal encodings, many of which showed scalability problems. After several sessions, we focused on a promising encoding called a “filament plot” (Fig. 5). Filament plots emanate from a common root, then proceed in a left-to-right direction aligned with the time sequence. Each filament represents the entire observation period for an ensemble member’s output attribute, with dots along the filament to indicate time stamps. To account for inter-member attribute variability, the curvature degree for the filament at each time step encodes the relative change from the previous rating, where upward rotation indicates value increases and vice versa for the downward rotation.

4 Contrail Group Detection and Tracking

4.1 Contrail Cluster Detection Example (Synthetic Data)

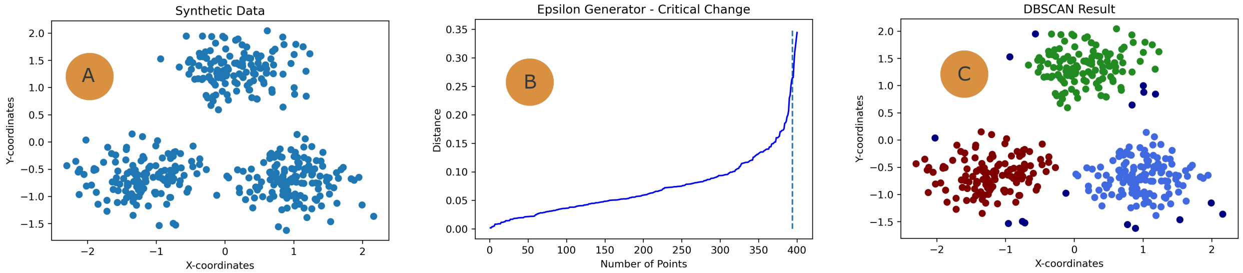

Figure 6: Example of Contrail Cluster Detection. (A) Synthetic 2D data corresponding to three groups, which need to be identified; (B) Calculating the Epsilon value - the critical change in the DBSCAN curve; (C) Result of the DBSCAN clustering algorithm - three clusters are generated based on the spatial density of the points.

4.2 Contrail Tracking Over Time

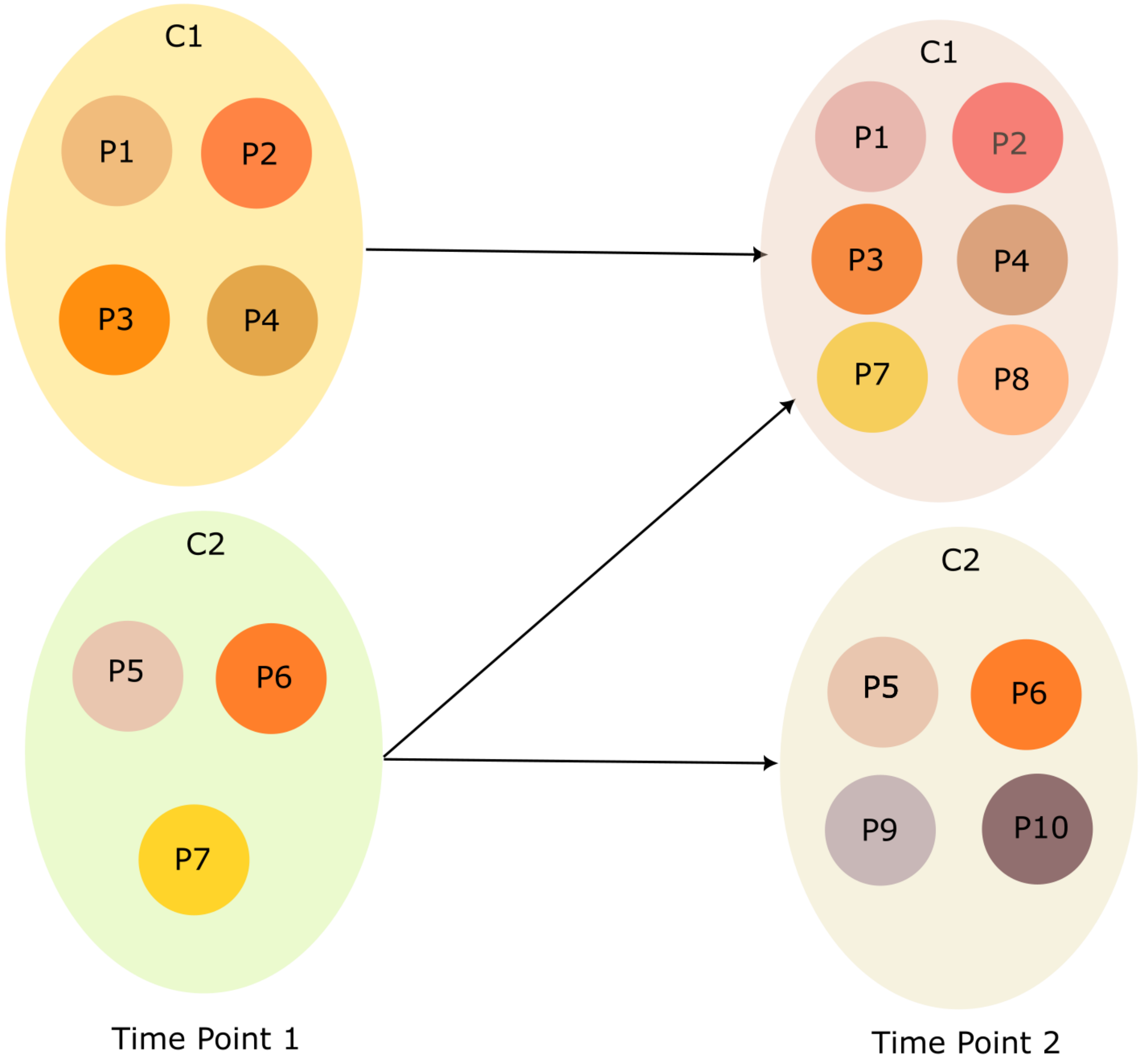

Figure 7: Example of Contrail Evolution over time. All particles of C1 in Time Point 1 are present in C1 in Time Point 2. Hence C1 in Time Point 1 merged into C1 in Time point 1, and a link between them shows the relationship. Particles in C2 in Time Point 1 are present in C1 and C2 in Time Point 2. Hence, C1 is merged into C1 and C2 in Time point 2. Links between them represent the relationship.
